# COVID-19 and pregnancy: A European study on pre- and post-infection medication use

**DOI:** 10.1007/s00228-024-03639-z

**Published:** 2024-02-12

**Authors:** Eimir Hurley, Benjamin P. Geisler, Angela Lupattelli, Beatriz Poblador-Plou, Régis Lassalle, Jérémy Jové, Marie-Agnes Bernard, Dunia Sakr, Gabriel Sanfélix-Gimeno, Francisco Sánchez-Saez, Clara L. Rodríguez-Bernal, Mònica Sabaté, Elena Ballarín, Cristina Aguilera, Sue Jordan, Daniel Thayer, Ian Farr, Saira Ahmed, Claudia Bartolini, Giorgio Limoncella, Olga Paoletti, Rosa Gini, Luigi A. Maglanoc, Elena Dudukina, Vera Ehrenstein, Ema Alsina, Tiago A. Vaz, Judit Riera-Arnau, Miriam C. J. M. Sturkenboom, Hedvig M. E. Nordeng

**Affiliations:** 1 PharmacoEpidemiology and Drug Safety Research Group, Department of Pharmacy, Faculty of Mathematics and Natural Sciences, University of Oslo, Oslo, Norway; 2 Massachusetts General Hospital, Harvard Medical School, Boston, MA USA; 3 EpiChron Research Group, Aragon Health Sciences Institute, Miguel Servet University Hospital, Saragossa, Spain; 4 Network for Research on Chronicity, Primary Care, and Health Promotion, Research Network on Health Services in Chronic Diseases, Institute of Health Carlos III, Madrid, Spain; 5 Bordeaux PharmacoEpi, Plateforme de recherche en Pharmaco-épidémiologie, Bordeaux, France; 6 Health Services Research and Pharmacoepidemiology Unit, Foundation for the Promotion of Health and Biomedical Research of Valencia Region, Valencia, Spain; 7 Department of Clinical Pharmacology, Vall d`Hebron Hospital Universitari, Vall Hebron Institut de Recerca, Barcelona, Spain; 8 Faculty of Medicine, Health and Life Science, Swansea University, Swansea, Wales UK; 9 Agenzia Regionale di Sanità della Toscana, Florence, Italy; 10 IT Department, Data Management, University of Oslo, Oslo, Norway; 11 Department of Clinical Epidemiology, Aarhus University and Aarhus University Hospital, Aarhus, Denmark; 12 Department of Data Science and Biostatistics, Julius Center for Health Sciences and Primary Care, University Medical Center Utrecht, Utrecht, The Netherlands; 13 Department of Child Health and Development, Norwegian Institute of Public Health, Oslo, Norway

**Keywords:** COVID-19, Pregnancy, Drug utilization study, Antithrombotic medications, Anti-bacterial agents, Steroids, Antiviral agents

## Abstract

**Purpose:**

The COVID-19 pandemic has impacted medication needs and prescribing practices, including those affecting pregnant women. Our goal was to investigate patterns of medication use among pregnant women with COVID-19, focusing on variations by trimester of infection and location.

**Methods:**

We conducted an observational study using six electronic healthcare databases from six European regions (Aragon/Spain; France; Norway; Tuscany, Italy; Valencia/Spain; and Wales/UK). The prevalence of primary care prescribing or dispensing was compared in the 30-day periods before and after a positive COVID-19 test or diagnosis.

**Results:**

The study included 294,126 pregnant women, of whom 8943 (3.0%) tested positive for, or were diagnosed with, COVID-19 during their pregnancy. A significantly higher use of antithrombotic medications was observed particularly after COVID-19 infection in the second and third trimesters. The highest increase was observed in the Valencia region where use of antithrombotic medications in the third trimester increased from 3.8% before COVID-19 to 61.9% after the infection. Increases in other countries were lower; for example, in Norway, the prevalence of antithrombotic medication use changed from around 1–2% before to around 6% after COVID-19 in the third trimester. Smaller and less consistent increases were observed in the use of other drug classes, such as antimicrobials and systemic corticosteroids.

**Conclusion:**

Our findings highlight the substantial impact of COVID-19 on primary care medication use among pregnant women, with a marked increase in the use of antithrombotic medications post-COVID-19. These results underscore the need for further research to understand the broader implications of these patterns on maternal and neonatal/fetal health outcomes.

**Supplementary Information:**

The online version contains supplementary material available at 10.1007/s00228-024-03639-z.

## Introduction

The coronavirus disease 2019 (COVID-19) pandemic has had a profound impact on global health and raised particular concerns for pregnant women [1]. Pregnancy involves physiological changes to the immune response and increases the risk of a more severe disease course with higher risks of intensive care admission, ventilatory support, and death, compared with women of with similar ages and risk factor profiles [2]. Furthermore, the use of medications during pregnancy is a complex issue, requiring careful consideration of the potential harms and benefits to both the mother and the fetus [3]. Understanding the patterns of medication use among pregnant women with COVID-19 is therefore of paramount importance for informing clinical practice and policy decisions [4].

Despite the importance of this issue, the existing literature is limited. Most studies to date have been based on small sample sizes and/or single-country data or self-reports, restricting the generalizability of the findings [5–7]. Moreover, there is a need for a more nuanced understanding of medication use among pregnant women with COVID-19, taking into account factors such as the trimester of pregnancy [8] and the severity of the infection [2, 9–11]. Guidelines for medication use in pregnant women with COVID-19 changed during the pandemic [4]. Existing medications were repurposed for use against COVID-19 but later found to be inefficacious, e.g., hydroxychloroquine or ivermectin; specific novel COVID-19 therapies or combinations only emerged later, e.g., nirmatrelvir-ritonavir or molnupiravir [12]. These gaps in knowledge highlight the need for comprehensive, multi-national studies that can provide a more complete picture of medication use in this context.

We therefore analyzed electronic health registers and other databases from six European regions with the aim of investigating medication use among pregnant women before and after a COVID-19 infection, stratified by trimester and disease severity. Our goal was to create insights into medication use patterns across different healthcare systems and stages of pregnancy, contributing to a more comprehensive understanding of the pharmacological management of COVID-19 in pregnant women.

## Methods

### Study design and data sources

As part of the multifaceted COVID-19 infectiOn aNd medicineS IN preGNancy (CONSIGN) project, we undertook a multinational observational cohort study using data from six regions in five countries across Europe. The data sources included linked prescription registers, primary care databases, birth registers, and administrative claims databases (collectively called databases) from Aragon, Spain; France; Norway; Tuscany, Italy; Valencia region, Spain; and Wales from the beginning of 2019 through the end of 2021. These databases provided information on patient demographics, medical history, outpatient and/or primary care prescribing or dispensing, pregnancies, and COVID-19 tests and/or diagnoses. Please see Supplementary Table 1 through 3 for details on the individual data sources contributing with data to this drug utilization study. The full study protocol is publicly available at the Zenodo open repository [13].

### Study population

We analyzed data from all women aged between 12 and 55 years, whose pregnancies overlapped with the COVID-19 pandemic (assumed start date of the pandemic was March 3, 2020; data available through December 31, 2021) and were registered in one of the participating databases. We used the ConcePTION pregnancy algorithm (v3.0) to detect pregnancies. This algorithm used various information sources in the databases and determined when each pregnancy started and ended [14]. All detected pregnancies linked to a clinically recognized outcome were included in our study, irrespective of the outcome of the pregnancy. The American College of Obstetricians and Gynecologists’ definition was used to determine trimesters [15]. For this paper, our study population consisted only of pregnant women with COVID-19.

### COVID-19 diagnosis

We identified women who tested positive for or were diagnosed with COVID-19 during pregnancy via records in surveillance systems or diagnostic codes, such as those in the ICD-10 and SCTSPA ontologies, and/or laboratory results in health care records. In all regions, except for France, a COVID-19 surveillance registry was utilized which allowed for the identification of a confirmed diagnosis. The French database, which was a 10% sample of the country’s data, was limited to inpatient data with ICD-10 codes of COVID-diagnoses. Hence, the French data included only pregnant women with a hospital admission for COVID-19 and not those who tested positive for COVID-19 in the community (see Supplementary Table 3).

COVID-19 disease severity was categorized based on whether or not the woman was admitted to hospital with a COVID-19 diagnosis. Information on intensive care unit admission or respiratory support was not known in many of the data sources, so no further categorization of severity level was possible. A hospitalization with COVID-19 was defined as any recording of a positive COVID-19 test (PCR or antigen test) or COVID-19 diagnosis in any of the diagnostic fields in hospital records (not just the principal diagnosis), within 4 weeks of initial COVID-19 positive test/diagnosis. If the COVID-19 test/positive diagnosis was within 2 days of the delivery date, these episodes were not classed as a hospitalized COVID-19 case. This was done to reduce the risk of misclassifying those COVID-19 cases coincidently detected during admission for delivery as hospitalization due to COVID-19. They were included in the analysis undertaken on the total cohort of individuals who tested positive for COVID-19 during their pregnancy but were excluded in the subgroup analysis on hospitalization status.

### Medication use

We examined the patterns of primary care and outpatient prescribing or dispensing of an extensive list of medication classes, as defined by the Anatomical Therapeutic Chemical Classification System, that were of particular relevance for COVID-19. Relevance of these medication groups relevant for COVID-19 was based on discussions within the research group and on relevant guidelines at the time of study conception. We also examined the use of specific medications within these classes, including the anticoagulants unfractionated heparin and enoxaparin, the anti-platelet agent acetylsalicylic acid, and the macrolide antibiotic azithromycin (see Supplementary Table 5). For each prescription/dispensing record, we used the prescription/dispensing date to reflect timing of actual medicine availability. We calculated the prevalence of primary care/outpatient medication use in the 30-day windows before and after a positive COVID-19 test or diagnosis. Medication use data during inpatient treatment periods was not available.

### Analysis

We performed descriptive analyses to summarize the characteristics of the study population and the prevalence of outpatient medication use. We used 95% confidence intervals derived from robust standard errors to quantify the uncertainty around the prevalence estimates. We stratified our analyses by trimester of pregnancy when infection occurred. Furthermore, we stratified hospitalized from non-hospitalized patients. Analyses were performed locally in each data partner’s site in R (R Foundation for Statistical Computing, Vienna, Austria) using data mapped to the ConcePTION common data model and data analytical pipeline [16].

### Ethics and data privacy

We pre-registered the present study in the European Union electronic Register of Post-Authorisation Studies (EU PAS Register Number: EUPAS39438). It was conducted in accordance with the ethical standards of the Declaration of Helsinki and was approved by the relevant ethical and governance review boards in each participating region; see Supplementary Table 4 for individual declarations. All data were pseudonymized to protect patient privacy and were handled and stored locally in each region in accordance with the General Data Protection Regulation.

## Results

The study cohort consisted of 294,126 pregnant women from the six European regions, with 8943 (3.0%) testing positive for or diagnosed with COVID-19 during their pregnancy. The proportion of pregnant women with COVID-19 varied by region, from 1.2% in Norway to 5.6% in Aragon, Spain (see Supplementary Figure depicting timing of COVID-19 infection in the cohort). The distribution of COVID-19 diagnoses across pregnancy trimesters also differed by region. In France, most infections occurred in the third trimester (90.7%) with a median gestational week (GW) of infection at GW40. In contrast, COVID-19 infection in the Valencia region of Spain and in Wales was more evenly distributed across trimesters, with the remaining regions of Tuscany, Aragon, and Norway falling in between the two ends of the spectrum but showing a greater proportion of trimester-3 infections (median GW at COVID-19 infection between GW22 and GW29) (see Table 1).

**Table 1:** Description of the multinational pregnancy cohort

**Region/Country**	**Data source**	**Data access provider**	**Total pregnancies (*N*)**	**COVID-19 positive test/diagnosis in pregnancy (*N* (%))**	**Diagnosis in trimester 1 (*N* (%))**	**Diagnosis in trimester 2 (*N* (%))**	**Diagnosis in trimester 3 (*N* (%))**	**Median gestational week of infection**
Aragon, Spain	PRECOVID Study and EpiChron Cohort	IACS	15,847	892 (5.6)	190 (21.3)	266 (29.8)	436 (48.9)	27
France	Système National des Données de Santé (SNDS)^a^	BPE	45,125	1069 (2.4)	7 (0.7)	92 (8.6)	970 (90.7)	40
Norway	Linked national registries (Norway)	UiO	87,038	1071 (1.2)	230 (21.5)	398 (37.2)	443 (41.4)	25
Tuscany, Italy	ARS database	ARS	41,415	992 (2.4)	207 (20.9)	277 (27.9)	508 (51.2)	29
Valencia, Spain	Valencia Integrated Database	FISABIO-HSRU	58,379	2978 (5.1)	941 (31.6)	856 (28.7)	1181 (39.7)	23
Wales	SAIL database (SAIL)	SWANSEA	46,322	1941 (4.2)	576 (29.7)	638 (32.9)	727 (37.5)	22
Total			294,126	8943 (3.0)	2151 (24.1)	2527 (28.3)	4265 (47.7)	

*ARS* Agenzia Regionale di Sanità della Toscana, *BPE* Bordeaux PharmacoEpi platform, *FISABIO-HSRU* Foundation for the Promotion of Health and Biomedical Research of Valencia Region - Health Services Research Unit, *IACS* Instituto Aragonés de Ciencias de la Salud, *SAIL* Secure Anonymised Information Linkage, *SNDS* Système National des Données de Santé, *SWANSEA* Swansea University, *UiO* University of Oslo

^a^Given the very broad inclusion criteria, a representative 1/10th sample of the full SNDS was used

**Fig. 1:**
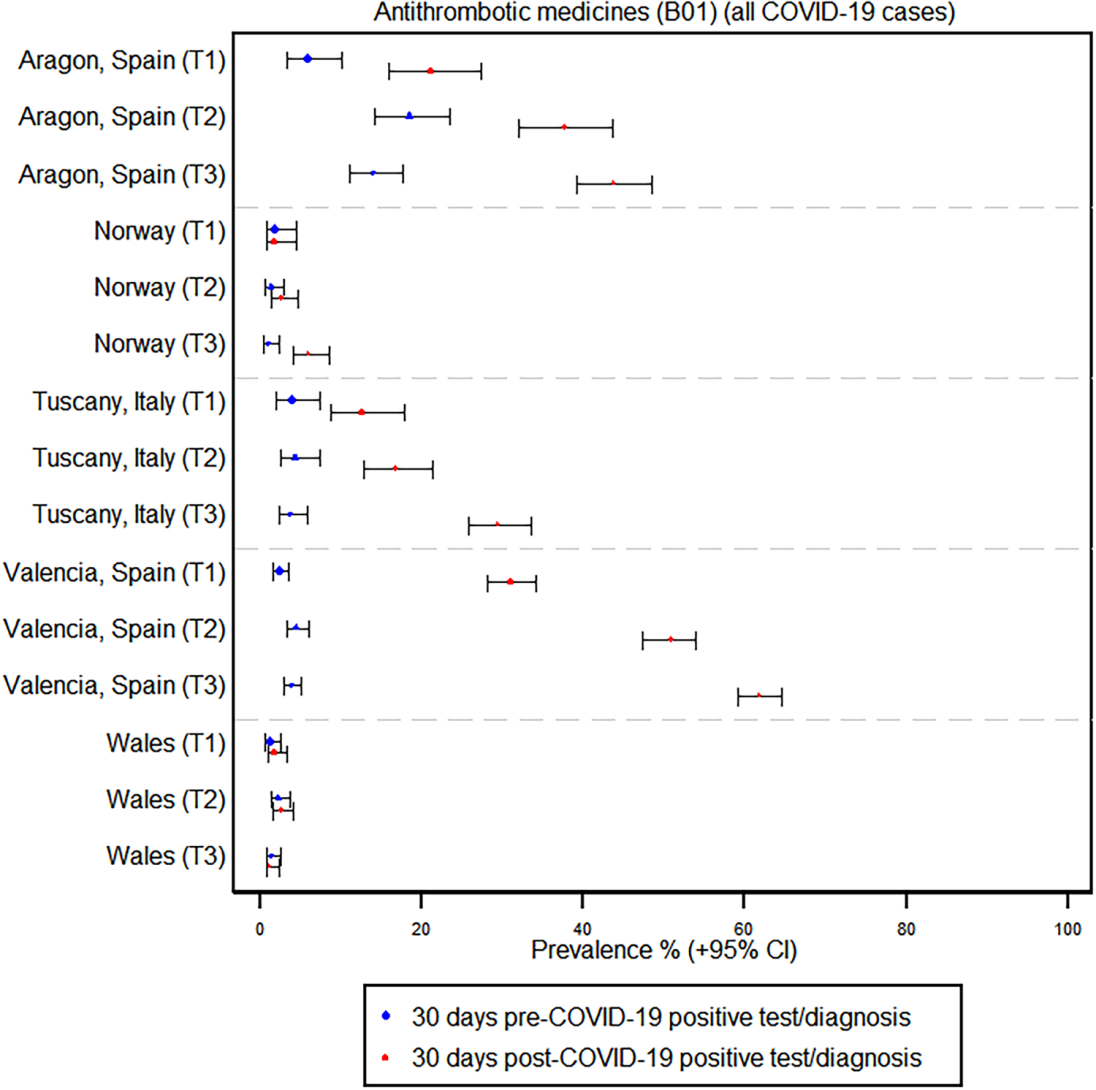
Prevalence of antithrombotic medication use before and after a positive COVID-19 test or diagnosis by region and trimester

The use of antithrombotic medications increased within the 30-day window following a positive COVID-19 test or diagnosis across all regions and trimesters. This increase was most pronounced in Valencia, Spain, where the prevalence of antithrombotic medication use rose from 2.3 to 31.0% in the first trimester, 4.4 to 50.7% in the second trimester, and 3.8 to 61.9% in the third trimester. Across all regions, the majority of antithrombotic medications were anticoagulants, accounting for 99.5% of all antithrombotic medication use in the third trimester in Valencia. In contrast, Norway, the country with the smallest antithrombotic medication use, had an increase from 1.3 to 2.5% in the second trimester and from 0.9 to 5.9% in the third trimester, with anticoagulants making up 91.5% of all antithrombotic medications used in the third trimester. The largest share of acetylsalicylic acid use was observed in the third trimester in Aragon, Spain (1.5% total use 30 days after a COVID-19 infection in the third trimester, corresponding to a 3.4% share of antithrombotic medications) (see Fig. 1 and Supplementary Tables 6–9).

The use of antithrombotic medications was similarly increased among the subset of women who were hospitalized due to or with COVID-19. For example, in the Valencia region of Spain, the prevalence of antithrombotic medication use in the third trimester increased from 2.4% before a COVID-19 hospitalization to 74.4% after. Enoxaparin was the most common antithrombotic medication, accounting for approximately 80% or higher of antithrombotic medication usage in all regions except Norway. The highest proportion of enoxaparin use was found in Aragon, Spain (> 97% of antithrombotic usage) (see Supplementary Table 6–9).

**Fig. 2:**
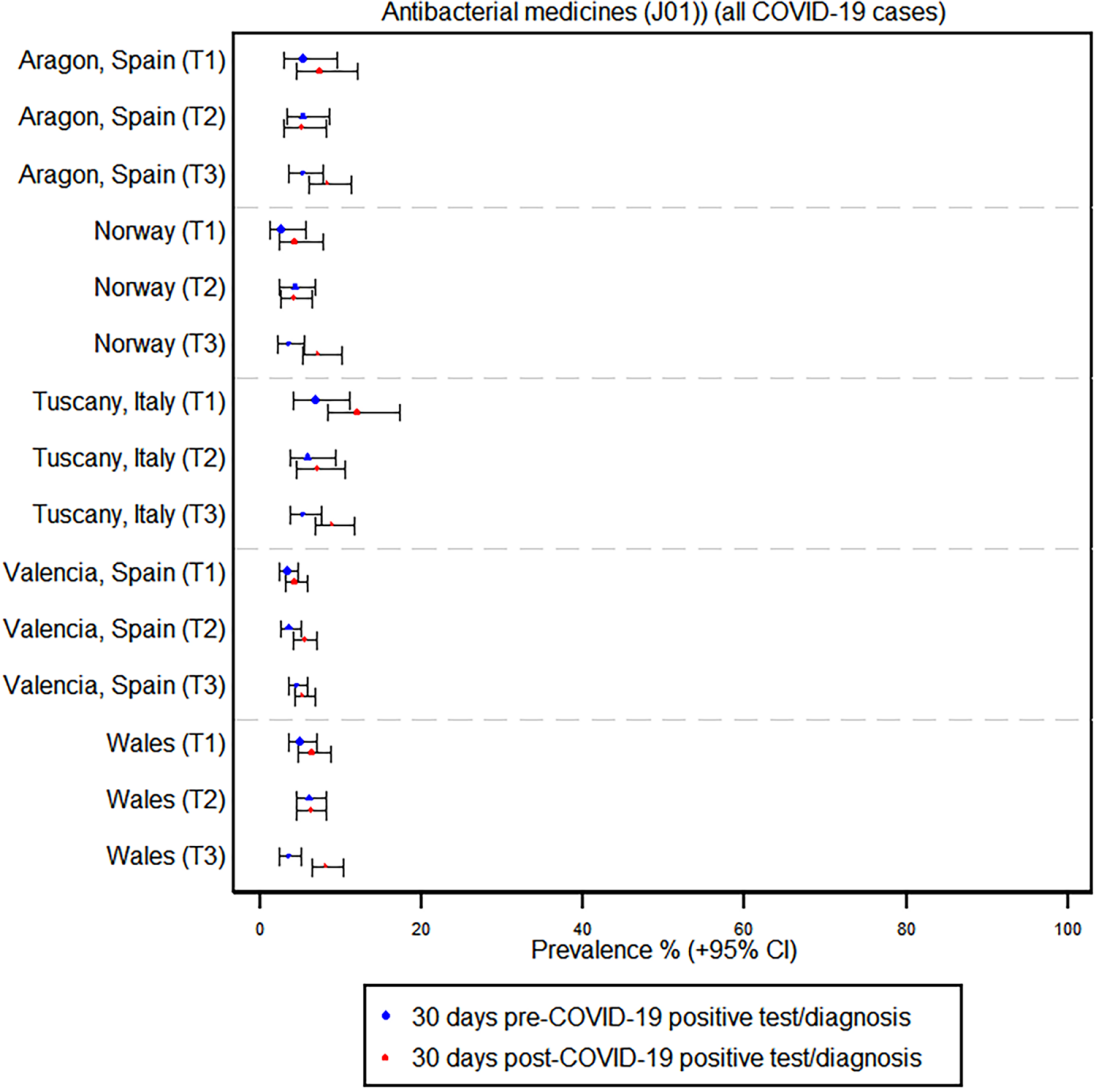
Prevalence of antibiotic use before and after a positive COVID-19 test or diagnosis by region and trimester

**Fig. 3:**
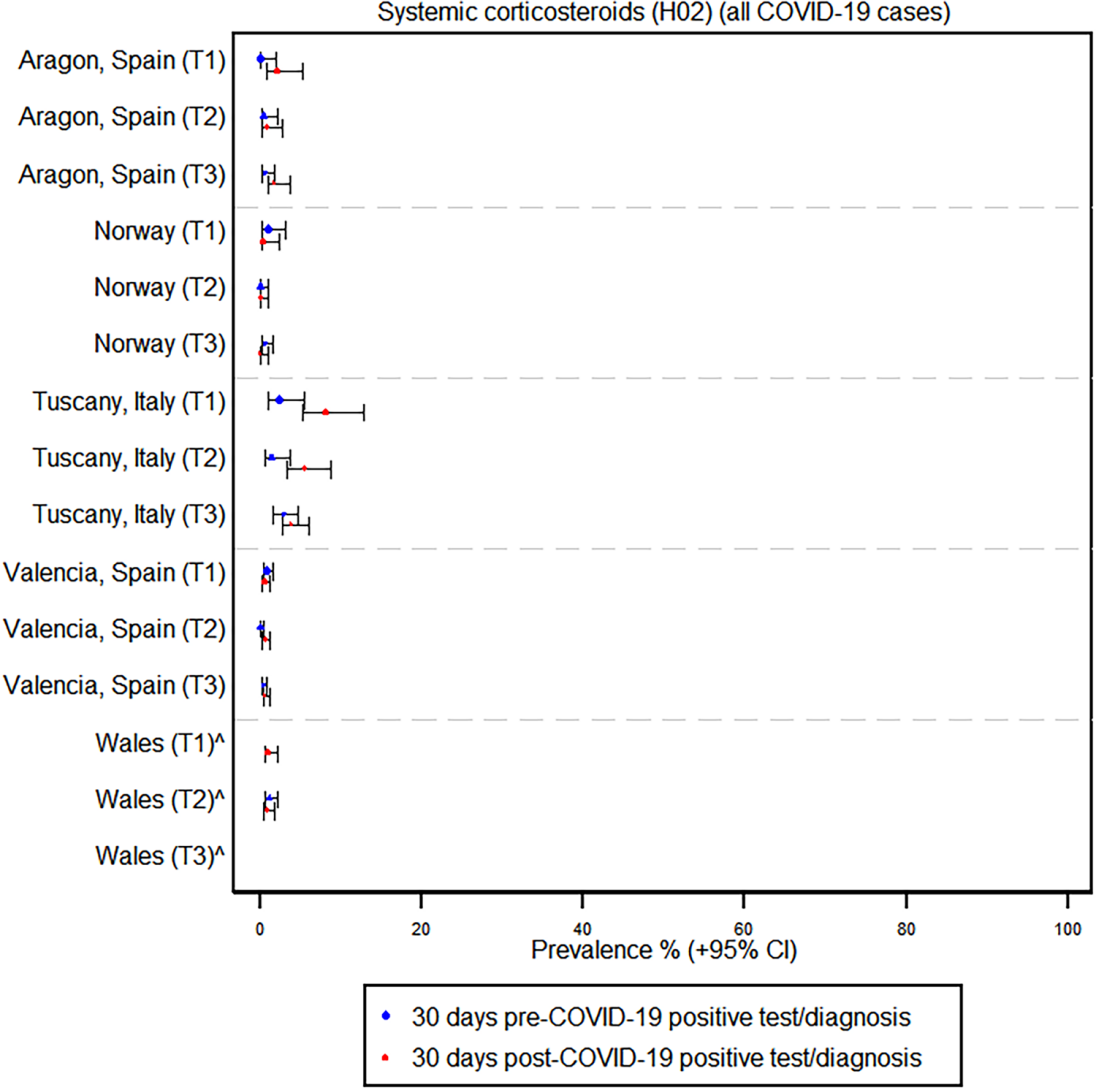
Prevalence of systemic corticosteroid use before and after a positive COVID-19 test or diagnosis by region and trimester

**Fig. 4:**
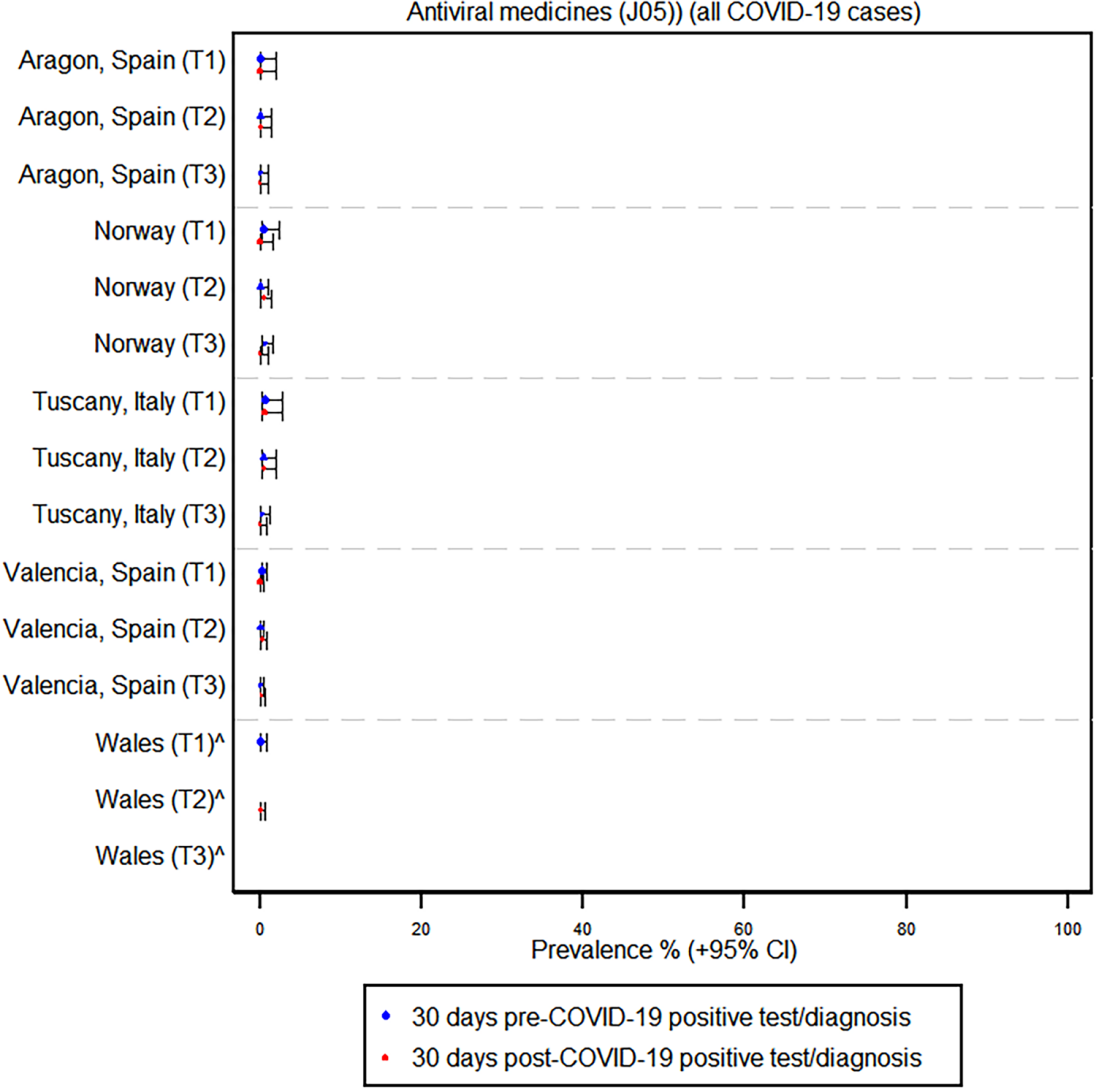
Prevalence of antiviral use before and after a positive COVID-19 test or diagnosis by region and trimester

In contrast, the use of antibiotics, corticosteroids, and antivirals did not show any large absolute increases across regions and trimesters following a COVID-19 infection. For instance, in Tuscany, Italy, the prevalence of azithromycin use increased from 2.4% before COVID-19 to 5.8% after infection in the first trimester, but there was no significant change in the second and third trimesters. Similarly, in Norway, the prevalence of azithromycin use in pregnancy remained at zero percent before and after COVID-19 across all trimesters. France, however, was the only region where there was a considerable increase in antibiotic prescriptions; the prevalence increased from 19.6 to 34.8% in trimester 2 and from 6.6 to 17.8% in trimester 3, whereas prescriptions decreased from 42.9 to 14.3% in trimester 1; the contribution of azithromycin to this increased antibiotic usage in France was negligible (see Fig. 2 and Supplementary Tables 10 and 11). The use of systemic corticosteroids and antivirals remained very low and relatively stable before and after COVID-19 infection across all trimesters. Corticosteroid use in France increased in trimester one infection, as well as in hospitalized cases in the Tuscany and Aragon regions (see Figs. 3 and 4 and Supplementary Table 12).

## Discussion

In this study, we found that few women had COVID-19 in pregnancy (3%), with the majority having the disease in the third trimester of pregnancy (48%). We observed regional variations in the use of antithrombotic medications among pregnant women with COVID-19. Notably, there were increases in the use of this drug class following a positive COVID-19 test or diagnosis in Tuscany, Italy; France (in the second and third trimesters); Valencia, Spain; and Aragon, Spain. Conversely, there were no or only very small increases in Wales and Norway. In contrast to antithrombotic medications, the use of antibiotics, antivirals, and corticosteroids did not show a consistent increase following a COVID-19 diagnosis.

Our findings have several important implications. The observed increase in antithrombotic medication use following a COVID-19 diagnosis, particularly in certain regions, may reflect clinicians’ response to the heightened risk of thromboembolic events associated with COVID-19 [17]. This could indicate an adaptation of clinical practice in light of then still emerging evidence during the pandemic [4]. Indeed, contemporary clinical COVID-19 guidelines universally recommend the administration of anticoagulants at prophylactic doses in hospitalized patients with severe disease [18–20]. The markedly higher use of antithrombotic medications, particularly enoxaparin, observed in Spain compared to other regions may have been influenced by national guidance recommending the use of low-molecular-weight heparins for all pregnant women with COVID-19 regardless of symptom severity or hospitalization status [21]. This contrasts with contemporary guidelines [18–20] and earlier guidance in some other countries [4] that recommended anticoagulants only for hospitalized patients or, in other instances, also for non-hospitalized and immobilized cases [22] or based on individual risk factors for venous thromboembolism [4]. Antithrombotic medications may not be indicated in mild-to-moderate COVID-19 except in anticoagulation of suspected or proven venous thromboembolism or when comorbidities are present [23]. The broader recommendations in Spain likely contributed to the substantial increase in antithrombotic medication use across trimesters and severity levels seen in the present study, compared with more selective use in regions following guidance targeting only severe disease. The observed variation in antithrombotic medication use across different regions may partly reflect baseline practices for managing venous thromboembolism and other comorbidities in pregnant women with COVID-19. While this could suggest a need for more uniform evidence-based guidelines, it also underscores the importance of considering regional clinical practices and existing comorbidity management strategies when interpreting these findings. Finally, the relatively stable use of antibiotics, corticosteroids, and antivirals suggests that these medications were not routinely used for COVID-19 treatment in pregnancy, possibly due to a perceived lower benefit vs. harm balance, specifically relating to potential harms to the fetus [24–26]. Taken together, these findings could inform future research on fetal and neonatal outcomes of pregnant women treated with medications for COVID-19.

Aside from two studies of self-reported medication use [6, 7], there is only one other published drug utilization study among pregnant women with COVID-19 [5], underscoring that this area remains under-investigated. The two self-reported surveys conducted during the pandemic reported that the most frequently reported drug categories during pregnancy were analgesics (28%), antihistamines (15%), antiacids (11%), antithrombotic medications (6%), and thyroid hormones (5%) in one study [6], and analgesics (14%), antihistamines (7%), antiacids (7%), antithrombotic medications (3%), and laxatives (3%) in the other [7]. These figures may be close to baseline utilization in pregnant women without COVID-19. In contrast, Westhoff et al. found, through an international registry, that the most commonly reported medications used among pregnant women with COVID-19 or those who had recently given birth, aside from analgesics, were azithromycin (13%), corticosteroids (4%), interferon (2%, driven by use in Russia), the antiviral oseltamivir (2%), chloroquine/hydroxychloroquine (2%), anticoagulants (2%), monoclonal antibodies (1%), and the antiviral remdesivir (> 0%) [5]. The study found that drug utilization patterns in pregnancy varied by country and severity of disease but not notably between trimesters of infection. A systematic review and meta-analysis of 62 observational studies (both case series and cohort studies) published between December 2019 and February 2021 included data on 31,016 pregnant women with confirmed COVID-19 [27]. The review found that among those studies that reported on the pharmacological treatments of COVID-19, approximately half of the pregnant women were given antibiotics, anticoagulants, and hydroxychloroquine, one in three was given antivirals, and nearly one in five was managed with either corticosteroids or immunotherapy [27]. However, the use of some of these medication groups may have been higher in severe cases, including in hospitalized patients. A preprint published by the OHDSI network of six databases consisting of electronic medical records and claims data from France, Spain, and the United States included 8598 pregnant women with COVID-19 (2,031 hospitalized) between January 2020 and June 2020 [28]. The ten most common inpatient treatments in the U.S. were systemic corticosteroids (29.6%), the low-molecular-weight heparin enoxaparin (24.0%), immunoglobulins (21.4%), famotidine (20.9%), azithromycin (18.1%), heparin (15.8%), ceftriaxone (7.9%), acetylsalicylic acid (7.0%), hydroxychloroquine (5.4%), and amoxicillin (3.5%). However, the study has not been peer-reviewed yet, and data were not presented on medication prescribed or dispensed in the outpatient setting. Additionally, variation in results between countries was neither presented nor discussed.

Our study distinguishes itself by its sample size and breadth of data. It is based on a large, multinational cohort of pregnant women, which enhances the generalizability of our findings across different healthcare systems and populations in Europe. Unlike many studies that rely on self-reported data, our study utilized prescription and/or dispensing data, providing a more objective measure of outpatient medication use. Furthermore, our study uniquely examined the use of various classes of medications, including antithrombotic medications, antibiotics, corticosteroids, and antivirals—offering a comprehensive view of medication patterns among pregnant women with COVID-19. In addition, our data are stratified by timing of COVID-19 infection in terms of the trimester of pregnancy and by hospitalization status. A larger proportion of positive COVID-19 tests and diagnoses occurred in the third trimester of pregnancy, which may be influenced by hospital policies. This was most evident in the French data, where COVID-19 diagnoses were exclusively based on inpatient hospital stays. This observation suggests that hospital testing protocols, especially during childbirth admissions, likely contributed to the higher incidence of COVID-19 diagnoses in the third trimester. The impact of these testing policies on medication use trends and their implications for understanding the management of COVID-19 in pregnant women warrants further consideration. Lastly, our study not only assessed the prevalence of medication use but also tracked changes in medication use before and after a COVID-19 infection, providing valuable insights into the impact of the pandemic on medication patterns in pregnancy.

However, our study is not without limitations. First, the observational nature of the study means we cannot establish a causal relationship between COVID-19 and increased antithrombotic medication use. However, the temporal relationship between a positive COVID-19 test or diagnosis and increased use of antithrombotic medications suggests a potential link that warrants clinical discussion and further investigation. Second, we did not have information on the severity of COVID-19 infection (aside from hospitalization status) or utilize data regarding the prevalence of comorbidities, both of which could influence medication use patterns. However, even without this information, our findings provide valuable insights into the real-world use of medications among pregnant women diagnosed with COVID-19. Third, we did not analyze drug utilization patterns stratified by pandemic period and/or variant since these varied by region. Of note, the main circulating SARS-CoV-2 variants during the study period were the original Wuhan strain, alpha, gamma, and delta, and—to a smaller degree—beta. Finally, the data sources varied, and we do not have information whether patients took their prescribed medications. For example, Wales relied solely on prescriptions written in general practice, including those under the direction of secondary- and tertiary-care clinicians, whereas all other regions used dispensing data, which might have captured more prescriptions from other sources including hospital-initiated prescriptions and specialty care. In France, the cohort was limited to a 10% sample of women with a hospital recording of COVID-19, which explains some of the different findings, e.g., most women had infections in the third trimester. However, despite these differences in data sources and without knowing whether medications were taken, the observed regional variations in prescriptions and/or dispensation highlight the need for consistent guidelines on the management of COVID-19 in pregnant women.

In conclusion, the present study confirms the feasibility of studying medication use patterns in a multinational cohort. Our results reveal significant regional variations, particularly in the use of antithrombotic medications among pregnant women with COVID-19. These variations underscore decision-makers’ and other stakeholders’ needs for further understanding of both the impetus behind these medication use patterns and the implications for maternal and perinatal health outcomes.

### Supplementary Information

Below is the link to the electronic supplementary material.

Supplementary file1 (DOCX 131 KB)

## Data Availability

Data supporting this study are included within the article and/or supporting materials. Information about data access should be directed to the individual data access provider.
