# Supplementary material for: COVID-19 and pregnancy: A European study on pre- and post-infection medication use
Source: Eur J Clin Pharmacol. 2024 Feb 12;80(5):707–16. doi: 10.1007/s00228-024-03639-z (PMC11001745; doi:10.1007/s00228-024-03639-z)
Supplement: Supplementary file 1 — Supplementary file1 (DOCX 131 KB) [file 228_2024_3639_MOESM1_ESM.docx]

**Supplementary Materials**

COVID-19 and Pregnancy: A European Study on Pre- and Post-Infection Medication Use

Eimir Hurley^1^([0000-0001-6776-1224](https://orcid.org/0000-0001-6776-1224)), Benjamin P. Geisler^1,2^([0000-0003-1704-6067](https://orcid.org/0000-0003-1704-6067)), Angela Lupattelli^1^([0000-0002-8787-3183](https://orcid.org/0000-0002-8787-3183)), Beatriz Poblador-Plou^3,4^([0000-0002-5119-5093](https://orcid.org/0000-0002-5119-5093)), Régis Lassalle^5^, Jérémy Jové^5^, Marie-Agnes Bernard^5^, Dunia Sakr^5^, Gabriel Sanfélix-Gimeno^6^([0000-0001-7098-4576](https://orcid.org/0000-0001-7098-4576)), Francisco Sánchez-Saez^6^([0000-0002-3011-9884](https://orcid.org/0000-0002-3011-9884)), Clara L. Rodríguez-Bernal^6^([0000-0003-2617-8635](https://orcid.org/0000-0003-2617-8635)), Mònica Sabaté^7^([0000-0001-6206-1085](https://orcid.org/0000-0001-6206-1085)), Elena Ballarín^7^([0000-0001-9786-6617](https://orcid.org/0000-0001-9786-6617)), Cristina Aguilera^7^([0000-0002-7985-7327](https://orcid.org/0000-0002-7985-7327)), Sue Jordan^8^([0000-0002-5691-2987](https://orcid.org/0000-0002-5691-2987)), Daniel Thayer^8^, Ian Farr^8^, Saira Ahmed^8^, Claudia Bartolini^9^([0000-0001-8630-4598](https://orcid.org/0000-0001-8630-4598)), Giorgio Limoncella^9^, Olga Paoletti^9^, Rosa Gini^9^([0000-0002-6250-877X](https://orcid.org/0000-0002-6250-877X)), Luigi A. Maglanoc^10^([0000-0003-2556-0779](https://orcid.org/0000-0003-2556-0779)), Elena Dudukina^11^([0000-0002-4238-049X](https://orcid.org/0000-0002-4238-049X)), Vera Ehrenstein^11^(0000-0002-3415-3254), Ema Alsina^12^, Tiago Andres Vaz^12^([0000-0003-3125-0662](https://orcid.org/0000-0003-3125-0662)), Judit Riera-Arnau^7,12^([0000-0001-7591-0218](https://orcid.org/0000-0001-7591-0218)), Miriam C.J.M. Sturkenboom^12^([0000-0003-1360-2388](https://orcid.org/0000-0003-1360-2388)), Hedvig M.E. Nordeng^1,13^([0000-0001-6361-2918](https://orcid.org/0000-0001-6361-2918))

1 PharmacoEpidemiology and Drug Safety Research Group, Department of Pharmacy, Faculty of Mathematics and Natural Sciences, University of Oslo, Norway

2 Massachusetts General Hospital/Harvard Medical School, Boston, MA, USA

3 EpiChron Research Group, Aragon Health Sciences Institute, Miguel Servet University Hospital, Zaragoza, Spain

4 Network for Research on Chronicity, Primary Care, and Health Promotion, Research Network on Health Services in Chronic Diseases, Institute of Health Carlos III, Madrid, Spain

5 Bordeaux PharmacoEpi, Plateforme de recherche en Pharmaco-épidémiologie, Bordeaux, France

6 Health Services Research and Pharmacoepidemiology Unit, Foundation for the Promotion of Health and Biomedical Research of Valencia Region, Valencia, Spain

7 Department of Clinical Pharmacology, Vall d'Hebron Hospital Universitari, Vall Hebron Institut de Recerca, Barcelona, Spain

8 Faculty of Medicine, Health and Life Science, Swansea University, Swansea, Wales, UK

9 Agenzia Regionale di Sanità della Toscana, Florence, Italy

10 IT Department, Data Management, University of Oslo, Oslo, Norway

11 Department of Clinical Epidemiology, Aarhus University and Aarhus University Hospital, Aarhus, Denmark

12 Department of Data Science and Biostatistics. Julius Center for Health Sciences and Primary Care. University Medical Center Utrecht, Utrecht, The Netherlands

13 Department of Child Health and Development, Norwegian Institute of Public Health, Oslo, Norway

Correspondence to:

Professor Hedvig M.E. Nordeng, Ph.D.

PharmacoEpidemiology and Drug Safety Research Group, Department of Pharmacy, Faculty of Mathematics and Natural Sciences, University of Oslo, Norway.

Email: [h.m.e.nordeng@farmasi.uio.no](mailto:h.m.e.nordeng@farmasi.uio.no)

Phone: +47 22 85 66 04

**Table of contents**

[Supplementary Table 1: Overview of data sources and end date of data available. 3](#_Toc146130850)

[Supplementary Table 2: Information on available data sources in the CONSIGN study. 4](#_Toc146130851)

[Supplementary Table 3: Data banks used to identify positive test or diagnosis for COVID-19. 5](#_Toc146130852)

[Supplementary Table 4: Ethical approval per data source. 6](#_Toc146130853)

[Supplementary Table 5: ATC-levels 2-3-4-5 of medicines of special relevance to COVID-19. 7](#_Toc146130854)

[Supplementary Table 6: Prevalence of antithrombotic medicines (B01) prescribed/dispensed in the 30 days pre- and post- COVID-19 positive test/diagnosis, to pregnant women, by trimester of pregnancy (all COVID-19 cases). 8](#_Toc146130855)

[Supplementary Table 7: Prevalence of antithrombotic medicines (B01) prescribed/dispensed in the 30 days pre- and post- COVID-19 positive test/diagnosis, to pregnant women, by trimester of pregnancy (not-hospitalized with COVID-19). 9](#_Toc146130856)

[Supplementary Table 8: Prevalence of antithrombotic medicines (B01) prescribed/dispensed in the 30 days pre- and post- COVID-19 positive test/diagnosis, to pregnant women, by trimester of pregnancy (hospitalized with COVID-19). 10](#_Toc146130857)

[Supplementary Table 9: Supplementary Table 9: Prevalence of antithrombotic medicine at the ATC- 3, 4, 5 level, in the 30-day window after COVID-19-positive test/diagnosis during the third trimester of pregnancy. 11](#_Toc146130858)

[Supplementary Table 10: Prevalence of antibacterial medicines (J01) prescribed/dispensed in the 30 days pre- and post- COVID-19 positive test/diagnosis, to pregnant women, by trimester of pregnancy (all COVID-19 cases). 12](#_Toc146130859)

[Supplementary Table 11: Prevalence of azithromycin (J01FA10) prescribed/dispensed in the 30 days pre- and post- COVID-19 positive test/diagnosis, to pregnant women, by trimester of pregnancy (all COVID-19 cases). 13](#_Toc146130860)

[Supplementary Table 12: Prevalence of corticosteroids for systemic use (H02) prescribed/dispensed in the 30 days pre- and post- COVID-19 positive test/diagnosis, to pregnant women, by trimester of pregnancy (all COVID-19 cases). 14](#_Toc146130861)

Supplementary Figure: Timing of COVID-19 infection in the pregnancy cohort 15

Supplementary Table 1: Overview of data sources and end date of data available.

| **Region/Country** | **Data source** | **Data Access provider** | **Estimated births per year** | **Total population** | **Type of data source** | **End date of data available** |
| --- | --- | --- | --- | --- | --- | --- |
| Aragon, Spain | PRECOVID Study and EpiChron Cohort | IACS | 10 000 | 1.3 million | Cohort | 2021-12-31 |
| 1/10^th^ representative sample, France^1^ | Système National des Données de Santé (SNDS)^1^ | BPE | 700 000  (70 000 in the 1/10th sample extracted) | 67 million  (6.7 million in the 1/10th sample extracted) | Health insurance | 2020-12-31 |
| Norway | Linked national registries (Norway) | UIO | 60 000 | 5.3 million | Record linkage | 2021-12-31 |
| Tuscany, Italy | ARS database | ARS | 25 000 | 3.6 million | Record linkage | 2021-12-31 |
| Valencia, Spain | Valencia Integrated Database (VID) | FISABIO-HSRU | 32 000 | 5 million | Record linkage | 2021-12-31 |
| Wales | SAIL database (SAIL) | SWANSEA | 29 000 | 3 million | Record linkage | 2021-12-31 |

Notes: ^1^Given the very broad inclusion criteria, a representative 1/10th sample of the full SNDS was used.

Abbreviations: ARS = Agenzia Regionale di Sanità della Toscana; BPE = Bordeaux PharmacoEpi platform; FISABIO-HSRU = Foundation for the Promotion of Health and Biomedical Research of Valencia Region - Health Services Research Unit; IACS = Instituto Aragonés de Ciencias de la Salud; SAIL = Secure Anonymised Information Linkage; SNDS = Système National des Données de Santé; SWANSEA = Swansea University; UiO = University of Oslo; VID = Valencia Integrated Database.

Supplementary Table 2: Information on available data sources in the CONSIGN study.

| **Region/Country** | **Data source** | **Data Access provider** | **Diagnoses recordings** | **Medicines** | **Medical birth registry** |
| --- | --- | --- | --- | --- | --- |
| Aragon, Spain | PRECOVID Study and EpiChron Cohort | IACS | GP, Hospital | Dispensing in community pharmacies | No |
| 1/10^th^ representative sample, France^1^ | Système National des Données de Santé (SNDS)^1^ | BPE | Hospital | Dispensing in community pharmacies or hospital pharmacies for outpatient use + expensive drugs dispensed in hospital (inpatient data) | No^2^ |
| Norway | Linked national registries (Norway) | UIO | GP, specialists,  Hospital | Dispensing in community pharmacies | Yes |
| Tuscany, Italy | ARS database | ARS | Hospital^3^ | Dispensing in community pharmacies | Yes |
| Valencia, Spain | Valencia Integrated Database (VID) | FISABIO-HSRU | GP, specialists,  Hospital | Dispensing and prescriptions (ambulatory care) | Yes |
| Wales | SAIL database (SAIL) | SWANSEA | GP, Hospital | Primary care prescribing for ~80% population^4^ | Yes |

Notes: ^1^Given the very broad inclusion criteria, a representative 1/10th sample of the full SNDS was used. ^2^Birth related outcomes are available through hospital discharge summary database, but there is not specific birth registry. ^3^Also emergency admissions, exemptions from co-payment for chronic conditions, access to mental healthcare. ^4^In Wales, ~80% primary care providers voluntarily supply medicines data to the databank. Any selection bias is due to healthcare providers, not subjects.

Abbreviations: ARS = Agenzia Regionale di Sanità della Toscan; BPE = Bordeaux PharmacoEpi platform; FISABIO-HSRU = Foundation for the Promotion of Health and Biomedical Research of Valencia Region - Health Services Research Unit; IACS = Instituto Aragonés de Ciencias de la Salud; SAIL = Secure Anonymised Information Linkage; SNDS = Système National des Données de Santé; SWANSEA = Swansea University; UiO = University of Oslo; VID = Valencia Integrated Database.

Supplementary Table 3: Data banks used to identify positive test or diagnosis for COVID-19.

| **Region/ Country** | **Data source** | **Data**  **Access provider** | **Type** | **Explanation** |
| --- | --- | --- | --- | --- |
| Aragon, Spain | PRECOVID Study and EpiChron Cohort | IACS | Registry developed for monitoring the evolution of the COVID-19 disease pandemic in the region of Aragon. | All PCR or antigen test results are recorded. |
| France | Système National des Données de Santé (SNDS) | BPE | Inpatient data (PMSI) with ICD10 codes of COVID-diagnoses. | No laboratory positive test result available |
| Norway | Linked national registries (Norway) | UIO | Norwegian surveillance system for communicable diseases (MSIS) | Laboratory-confirmed positive test |
| Tuscany, Italy | ARS database | ARS | Registry COVID-19 with ICD9 codes of COVID-diagnoses. | This is the official surveillance system of the pandemic. Several variables are collected on the case and during the first year of the pandemic the variables were updated during the disease. |
| Valencia, Spain | Valencia Integrated Database (VID) | FISABIO-HSRU | RedMIVA (Microbiological Surveillance Network of the Valencian Community) and ICD10CM (ICD-10-ES) codes | All PCR or antigen test results are recorded |
| Wales | SAIL database (SAIL) | SWANSEA | COVID-19 test results dataset available from [healthdatagateway.org](https://web.www.healthdatagateway.org/dataset/594cfe55-96e3-45ff-874c-2c0006eeb881) | All test results and symptom trackers |

Abbreviations: ARS = Agenzia Regionale di Sanità della Toscana; BPE = Bordeaux PharmacoEpi platform; FISABIO-HSRU = Foundation for the Promotion of Health and Biomedical Research of Valencia Region - Health Services Research Unit; IACS = Instituto Aragones de Ciencias de la Salud; SAIL = Secure Anonymised Information Linkage; SNDS = Système National des Données de Santé; SWANSEA = Swansea University; UiO = University of Oslo; VID = Valencia Integrated Database.

Supplementary Table 4: Ethical approval per data source.

| **Region/  Country** | **Data source** | **Data  Access provider** | **Details of ethical approval** |
| --- | --- | --- | --- |
| Aragon, Spain | PRECOVID Study and EpiChron Cohort | IACS | The Clinical Research Ethics Committee of Aragón (CEICA) approved this study (Research protocol PI21/029) and waived the requirement to obtain informed consent from patients given the epidemiological nature of the project and the use of anonymized data. |
| France | Système National des Données de Santé (SNDS) | BPE | Approval for a EU-COVID-19 project was obtained from Ethics and Scientific Committee (CESREES) and French Data protection authority (CNIL) (approval number: DR-2020-371) |
| Norway | Linked national registries (Norway) | UiO | Ethical approval for the Norwegian data in this study was obtained from The Regional Committee for Research Ethics (EU-COVID-19, approval number: 155294 /REK Nord) and the Data Protection Officer at the University of Oslo (approval number 523275). |
| Tuscany, Italy | ARS database | ARS | During the study period, ARS Toscana had approval by the Regional Council of Tuscany to access and analyse the data. |
| Valencia, Spain | Valencia Integrated Database (VID) | FISABIO-HSRU | Ethical approval for VID data (Valencia region, Spain) was obtained from the Ethics Committee on Medicines Research of Hospital Clinico Universitario de Valencia (approval number: 180/20) and the data access approval by Data Access Commission of the Valencia region (approval number: SD2270). |
| Wales | SAIL database (SAIL) | SWANSEA | The Secure Anonymised Information Linkage [SAIL] Databank Information Governance Review Panel [IGRP] approved the study on behalf of the National Research Ethics Service, Wales on 25th January 2020. Data were irrevocably anonymised and obtained with permission of the relevant Caldicott Guardian and Data Protection Officer. The project has been given a SAIL project reference number of 0823.  Wales only provided data on live and still births and EUROCAT births. This is due to governance restrictions |

Abbreviations: ARS = Agenzia Regionale di Sanita’ della Toscana; BPE = Bordeaux PharmacoEpi platform; FISABIO-HSRU = Foundation for the Promotion of Health and Biomedical Research of Valencia Region - Health Services Research Unit; IACS = Instituto Aragonés de Ciencias de la Salud; SAIL = Secure Anonymised Information Linkage; SNDS = Système National des Données de Santé; SWANSEA = Swansea University; UiO = University of Oslo; VID = Valencia Integrated Database.

Supplementary Table 5: ATC-levels 2-3-4-5 of medicines of special relevance to COVID-19.

| **ATC level 2 name** | **ATC level 2 code** | **ATC level 3 name** | **ATC level 3 code** | **ATC level 4 name** | **ATC level 4 code** | **ATC level 5 name** | **ATC level 5 code** |
| --- | --- | --- | --- | --- | --- | --- | --- |
| Antithrombotic agents | B01 | Antithrombotic agents | B01A | Heparin group | B01AB | Enoxaparin (low molecular weight heparin) | B01AB05 |
|  |  |  |  |  |  | Heparin (unfractionated) | B01AB01 |
|  |  |  |  | Platelet aggregation inhibitors excl. heparin | B01AC | Acetylsalicylic acid | B01AC06 |
| Corticosteroids for systemic use | H02 |  |  | Glucocorticoids | H02AB |  |  |
| Antibacterials for systemic use | J01 |  |  |  |  | Azithromycin | J01FA10 |
| Antivirals for systemic use | J05 |  |  |  |  | Remdesivir | J05AB16 |
|  |  |  |  |  |  | Lopinavir-ritonavir | J05AR10 |
|  |  |  |  |  |  | Oseltamivir | J05AH02 |
|  |  |  |  |  |  | Ribavirin | J05AP01 |
|  |  |  |  |  |  | Favipiravir | J05AX27 |
| Immune sera and globulins | J06 |  |  |  |  |  |  |
| Immunostimulants | L03 |  |  |  |  |  |  |
| Immunosuppressants | L04 |  |  |  |  |  |  |
| Anti-inflammatory drugs | M01 |  |  |  |  |  |  |
| Analgesics | N02 |  |  |  |  |  |  |
| Anthelmintics |  |  |  |  |  | Ivermectin | P02CF01 |
| Medicines for obstructive airway disease | R03 |  |  |  |  |  |  |

Supplementary Table 6: Prevalence of antithrombotic medicines (B01) prescribed/dispensed in the 30 days pre- and post- COVID-19 positive test/diagnosis, to pregnant women, by trimester of pregnancy (all COVID-19 cases).

|  | COVID-19 test/diagnoses in the first trimester | | COVID-19 test/diagnoses in the second trimester | | COVID-19 test/diagnoses in the third trimester | |
| --- | --- | --- | --- | --- | --- | --- |
| Region/Country | 30 days pre- | 30 days post- | 30 days pre- | 30 days post- | 30 days pre- | 30 days post- |
|  | % (95% CI) | % (95% CI) | % (95% CI) | % (95% CI) | % (95% CI) | % (95% CI) |
| Aragon, Spain | 5.2 (2.9-9.1) | 20.8 (15.8-26.7) | 17.9 (13.9-22.8) | 37.5 (32.1-43.3) | 14.7 (11.8-18.2) | 44.7 (40.2-49.3) |
| 1/10^th^ sample, France^1^ | 0 (0.0-35.4) | 14.3 (2.6-51.3) | 3.3 (1.1-9.2) | 6.5 (3-13.5) | 1.9 (1.2-2.9) | 22.8 (20.3-25.5) |
| Norway | 1.7 (0.7-4.4) | 1.7 (0.7-4.4) | 1.3 (0.5-2.9) | 2.5 (1.4-4.6) | 0.9 (0.4-2.3) | 5.9 (4-8.5) |
| Tuscany, Italy | 3.9 (2-7.4) | 12.6 (8.7-17.8) | 4.3 (2.5-7.4) | 16.6 (12.7-21.4) | 3.7 (2.4-5.8) | 29.5 (25.7-33.6) |
| Valencia, Spain | 2.3 (1.5-3.5) | 31 (28.2-34.1) | 4.4 (3.3-6) | 50.7 (47.4-54) | 3.8 (2.9-5.1) | 61.9 (59.1-64.6) |
| Wales | 1.2 (0.6-2.5) | 1.7 (0.9-3.2) | 2.2 (1.3-3.6) | 2.5 (1.5-4.0) | 1.4 (0.7-2.5) | 1.2 (0.7-2.3) |

Note: ^1^In France, COVID-19 cases were identified based on admission to hospital with a COVID-19 diagnosis.

Supplementary Table 7: Prevalence of antithrombotic medicines (B01) prescribed/dispensed in the 30 days pre- and post- COVID-19 positive test/diagnosis, to pregnant women, by trimester of pregnancy (not-hospitalized with COVID-19).

|  | COVID-19 test/diagnoses in the first trimester | | COVID-19 test/diagnoses in the second trimester | | COVID-19 test/diagnoses in the third trimester | |
| --- | --- | --- | --- | --- | --- | --- |
| Region/Country | 30 days pre- | 30 days post- | 30 days pre- | 30 days post- | 30 days pre- | 30 days post- |
|  | % (95% CI) | % (95% CI) | % (95% CI) | % (95% CI) | % (95% CI) | % (95% CI) |
| Aragon, Spain | 5.6 (3.2-9.8) | 19.5 (14.5-25.6) | 18 (13.8-23) | 35.6 (30.1-41.5) | 18.5 (13.9-24.1) | 33.3 (27.5-39.8) |
| 1/10^th^ sample, France^1^ | NA | NA | NA | NA | NA | NA |
| Norway | 1.8 (0.7-4.6) | 1.8 (0.7-4.6) | 1.1 (0.4-2.8) | 0.8 (0.3-2.4) | 0.3 (0.1-1.9) | 2.3 (1.1-4.7) |
| Tuscany, Italy | 4.4 (2.3-8.5) | 10.5 (6.8-15.8) | 4.2 (2.4-7.4) | 14.6 (10.8-19.4) | 2.7 (1.4-5.2) | 21.3 (17-26.3) |
| Valencia, Spain | 2.3 (1.5-3.5) | 31.3 (28.3-34.3) | 4.3 (3.1-5.9) | 50.4 (47-53.8) | 4.5 (3.2-6.2) | 61.8 (58.3-65.2) |
| Wales^2^ | * | * | * | 13.9 (6.1-28.7) | * | * |

Note: ^1^In France, COVID-19 cases were identified based on admission to hospital with a COVID-19 diagnosis. Hence there are no cases classed as non-hospitalized.

^2^Wales cannot release numbers between 1 and 4 either directly or in a cell that would disclose a number 1-4 elsewhere.

Supplementary Table 8: Prevalence of antithrombotic medicines (B01) prescribed/dispensed in the 30 days pre- and post- COVID-19 positive test/diagnosis, to pregnant women, by trimester of pregnancy (hospitalized with COVID-19^1^).

|  | COVID-19 test/diagnoses in the first trimester | | COVID-19 test/diagnoses in the second trimester | | COVID-19 test/diagnoses in the third trimester | |
| --- | --- | --- | --- | --- | --- | --- |
| Region/Country | 30 days pre- | 30 days post- | 30 days pre- | 30 days post- | 30 days pre- | 30 days post- |
|  | % (95% CI) | % (95% CI) | % (95% CI) | % (95% CI) | % (95% CI) | % (95% CI) |
| Aragon, Spain | 0 (0.0-24.2) | 41.7 (19.3-68) | 16.7 (5.8-39.2) | 66.7 (43.7-83.7) | 24.3 (16-35.2) | 83.8 (73.8-90.5) |
| 1/10^th^ sample, France^2^ | 0 (0.0-35.4) | 14.3 (2.6-51.3) | 3.3 (1.1-9.2) | 6.5 (3-13.5) | 3.1 (1.5-6.3) | 25.9 (20.6-32) |
| Norway | 0 (0.0-22.8) | 0 (0.0-22.8) | 2.6 (0.5-13.2) | 17.9 (9-32.7) | 2.9 (1.0-8.1) | 15.4 (9.7-23.5) |
| Tuscany, Italy | 0 (0.0-29.9) | 66.7 (35.4-87.9) | 5.9 (1.0-27.0) | 47.1 (26.2-69.0) | 7.5 (3.9-14.2) | 50 (40.6-59.4) |
| Valencia, Spain | 0 (0.0-39.0) | 50 (18.8-81.2) | 7.7 (1.4-33.3) | 61.5 (35.5-82.3) | 2.4 (0.7-8.5) | 74.4 (64-82.6) |
| Wales^3^ | * | * | * | 1.8 (1.0-3.2) | * | * |

Note: ^1^Excludes women with COVID-19 within two days of delivery to reduce the risk of misclassifying those COVID-19 cases coincidently detected during admission for delivery as hospitalization due to COVID-19. ^2^In France, COVID-19 cases were identified based on admission to hospital with a COVID-19 diagnosis. ^3^Wales cannot release numbers between 1 and 4 either directly or in a cell that would disclose a number 1-4 elsewhere.

Supplementary Table 9: Prevalence of antithrombotic medicine at the ATC- 3, 4, 5 level, in the 30-day window after COVID-19-positive test/diagnosis during the third trimester of pregnancy.

| ATC –level name (code) | Antithrombotic medicines (B01A) | Heparin group (B01AB) | Heparin (Unfractionated) (B01AB01) | Enoxaparin  (B01AB05) | Acetylsalicylic acid (B01AC06) |
| --- | --- | --- | --- | --- | --- |
| Region/Country^1^ | % (95% CI) | % (95% CI) | % (95% CI) | % (95% CI) | % (95% CI) |
| **All COVID-19 cases** | | | | | |
| Aragon, Spain | 44.7 (40.2-49.3) | 43.2 (38.8-47.7) | 0 (0.0-0.8) | 42.1 (37.7-46.7) | 1.5 (0.7-3.1) |
| 1/10^th^ sample, France^2^ | 22.8 (20.3-25.5) | 22.6 (20.1-25.3) | 0 (0.0-0.4) | 20.0 (17.6-22.6) | 0.5 (0.2-1.2) |
| Norway | 5.9 (4.0-8.5) | 5.4 (3.7-7.9) | 0 (0.0-0.9) | 2.5 (1.4-4.4) | 0.5 (0.1-1.6) |
| Tuscany, Italy | 29.5 (25.7-33.6) | 29.1 (25.4-33.2) | 0 (0.0-0.8) | 22.6 (19.2-26.5) | 0.6 (0.2-1.7) |
| Valencia, Spain | 61.9 (59.1-64.6) | 61.6 (58.8-64.3) | 0 (0.0-0.3) | 48.7 (45.8-51.5) | 1.4 (0.9-2.3) |
|  |  |  |  |  |  |
| **Not-hospitalized with COVID-19** | | | | | |
| Aragon, Spain | 33.3 (27.5-39.8) | 31.5 (25.8-37.9) | 0 (0.0-1.7) | 31.5 (25.8-37.9) | 2.3 (1.0-5.2) |
| 1/10^th^ sample, France^2^ | NA | NA | NA | NA | NA |
| Norway | 2.3 (1.1-4.7) | 1.7 (0.7-3.8) | 0 (0.0-1.3) | 0.7 (0.2-2.4) | 0.7 (0.2-2.4) |
| Tuscany, Italy | 21.3 (17-26.3) | 20.6 (16.4-25.6) | 0 (0.0-1.3) | 14.9 (11.3-19.4) | 0.7 (0.2-2.4) |
| Valencia, Spain | 61.8 (58.3-65.2) | 61.4 (57.9-64.8) | 0 (0.0-0.5) | 49.9 (46.3-53.4) | 1.9 (1.1-3.1) |
|  |  |  |  |  |  |
| **Hospitalized with COVID-19^3^** | | | | | |
| Aragon, Spain | 83.8 (73.8-90.5) | 83.8 (73.8-90.5) | 0 (0.0-4.9) | 77.0 (66.3-85.1) | 2.7 (0.7-9.3) |
| 1/10^th^ sample, France^2^ | 25.9 (20.6-32.0) | 25.4 (20.2-31.5) | 0 (0.0-1.7) | 22.8 (17.8-28.7) | 1.3 (0.5-3.9) |
| Norway | 15.4 (9.7-23.5) | 15.4 (9.7-23.5) | 0 (0.0-3.6) | 7.7 (3.9-14.4) | 0 (0.0-3.6) |
| Tuscany, Italy | 50.0 (40.6-59.4) | 50.0 (40.6-59.4) | 0 (0.0-3.5) | 44.3 (35.2-53.8) | 0.9 (0.2-5.2) |
| Valencia, Spain | 74.4 (64-82.6) | 73.2 (62.7-81.6) | 0 (0.0-4.5) | 58.5 (47.7-68.6) | 2.4 (0.7-8.5) |

Note: ^1^Wales cannot release numbers between 1 and 4 either directly or in a cell that would disclose a number 1-4 elsewhere.^2^In France, COVID-19 cases were identified based on admission to hospital with a COVID-19 diagnosis. Hence, there are no cases classed as not hospitalized. ^3^Excludes women with COVID-19 within two days of delivery to reduce the risk of misclassifying those COVID-19 cases coincidently detected during admission for delivery as hospitalization due to COVID-19.

Supplementary Table 10: Prevalence of antibacterial medicines (J01) prescribed/dispensed in the 30 days pre- and post- COVID-19 positive test/diagnosis, to pregnant women, by trimester of pregnancy (all COVID-19 cases).

|  | COVID-19 test/diagnoses in the first trimester | | COVID-19 test/diagnoses in the second trimester | | COVID-19 test/diagnoses in the third trimester | |
| --- | --- | --- | --- | --- | --- | --- |
| Region/Country | 30 days pre- | 30 days post- | 30 days pre- | 30 days post- | 30 days pre- | 30 days post- |
|  | % (95% CI) | % (95% CI) | % (95% CI) | % (95% CI) | % (95% CI) | % (95% CI) |
| Aragon, Spain | 5.7 (3.3-9.6) | 7.5 (4.7-11.9) | 5.6 (3.5-8.9) | 5.3 (3.2-8.5) | 5.2 (3.5-7.6) | 8.4 (6.2-11.3) |
| 1/10th sample, France^1^ | 42.9 (15.8-75.0) | 14.3 (2.6-51.3) | 19.6 (12.7-28.8) | 34.8 (25.8-44.9) | 6.6 (5.2-8.3) | 17.8 (15.6-20.4) |
| Norway | 2.6 (1.2-5.6) | 4.3 (2.4-7.8) | 4.3 (2.7-6.7) | 4 (2.5-6.4) | 3.4 (2.1-5.5) | 7.2 (5.2-10.0) |
| Tuscany, Italy | 6.8 (4.1-11.0) | 12.1 (8.3-17.2) | 5.8 (3.6-9.2) | 6.9 (4.4-10.5) | 5.3 (3.7-7.6) | 8.9 (6.7-11.6) |
| Valencia, Spain | 3.3 (2.3-4.6) | 4.3 (3.1-5.7) | 3.5 (2.5-5.0) | 5.3 (4.0-7.0) | 4.5 (3.4-5.8) | 5.3 (4.2-6.8) |
| Wales | 4.9 (3.4-6.9) | 6.4 (4.7-8.7) | 6.0 (4.4-8.1) | 6.1 (4.5-8.2) | 3.4 (2.3-5.0) | 8.1 (6.3-10.3) |

Notes: ^1^In France, COVID-19 cases were identified based on admission to hospital with a COVID-19 diagnosis.

Supplementary Table 11: Prevalence of azithromycin (J01FA10) prescribed/dispensed in the 30 days pre- and post- COVID-19 positive test/diagnosis, to pregnant women, by trimester of pregnancy (all COVID-19 cases).

|  | COVID-19 test/diagnoses in the first trimester | | COVID-19 test/diagnoses in the second trimester | | COVID-19 test/diagnoses in the third trimester | |
| --- | --- | --- | --- | --- | --- | --- |
| Region/Country | 30 days pre- | 30 days post- | 30 days pre- | 30 days post- | 30 days pre- | 30 days post- |
|  | % (95% CI) | % (95% CI) | % (95% CI) | % (95% CI) | % (95% CI) | % (95% CI) |
| Aragon, Spain | 0.5 (0.1-2.6) | 0 (0.0-1.8) | 0 (0.0-1.3) | 0.4 (0.1-2.0) | 0.4 (0.1-1.6) | 0.2 (0.0-1.2) |
| 1/10^th^ sample, France^1^ | 0 (0.0-35.4) | 0 (0.0-35.4) | 0 (0.0-4.0) | 3.3 (1.1-9.2) | 0 (0.0-0.4) | 0.8 (0.4-1.6) |
| Norway | 0 (0.0-1.6) | 0 (0.0-1.6) | 0 (0.0-1.0) | 0 (0.0-1.0) | 0 (0.0-0.9) | 0 (0.0-0.9) |
| Tuscany, Italy | 2.4 (1.0-5.5) | 5.8 (3.3-9.9) | 1.1 (0.4-3.1) | 1.8 (0.8-4.2) | 0.8 (0.3-2) | 1.8 (0.9-3.3) |
| Valencia, Spain | 0.2 (0.1-0.8) | 1 (0.5-1.8) | 0.1 (0-0.7) | 0.2 (0.1-0.8) | 0 (0.0-0.3) | 0.1 (0.0-0.5) |

Note: ^1^In France, COVID-19 cases were identified based on admission to hospital with a COVID-19 diagnosis.

Supplementary Table 12: Prevalence of corticosteroids for systemic use (H02) prescribed/dispensed in the 30 days pre- and post- COVID-19 positive test/diagnosis, to pregnant women, by trimester of pregnancy (all COVID-19 cases).

|  | COVID-19 test/diagnoses in the first trimester | | COVID-19 test/diagnoses in the second trimester | | COVID-19 test/diagnoses in the third trimester | |
| --- | --- | --- | --- | --- | --- | --- |
| Region/Country | 30 days pre- | 30 days post- | 30 days pre- | 30 days post- | 30 days pre- | 30 days post- |
|  | % (95% CI) | % (95% CI) | % (95% CI) | % (95% CI) | % (95% CI) | % (95% CI) |
| Aragon, Spain | 0 (0.0-1.8) | 2.4 (1.0-5.4) | 0.4 (0.1-2.0) | 0.7 (0.2-2.5) | 0.9 (0.3-2.2) | 1.9 (1-3.7) |
| 1/10^th^ sample, France^1^ | 0 (0.0-35.4) | 14.3 (2.6-51.3) | 5.4 (2.3-12.1) | 4.3 (1.7-10.7) | 2.1 (1.3-3.2) | 1.3 (0.8-2.3) |
| Norway | 0.9 (0.2-3.1) | 0.4 (0.1-2.4) | 0 (0.0-1.0) | 0 (0.0-1.0) | 0.5 (0.1-1.6) | 0 (0.0-0.9) |
| Tuscany, Italy | 2.4 (1-5.5.0) | 8.2 (5.2-12.8) | 1.4 (0.6-3.7) | 5.4 (3.3-8.7) | 2.8 (1.6-4.6) | 3.9 (2.6-6.0) |
| Valencia, Spain | 0.7 (0.4-1.5) | 0.5 (0.2-1.2) | 0 (0.0-0.4) | 0.5 (0.2-1.2) | 0.3 (0.1-0.7) | 0.6 (0.3-1.2) |
| Wales^2^ | * | 1 (0.5-2.3) | 1.1 (0.5-2.2) | 0.8 (0.3-1.8) | * | * |

Notes: ^1^In France, COVID-19 cases were identified based on admission to hospital with a COVID-19 diagnosis. ^2^Wales cannot release numbers between 1 and 4 either directly or in a cell that would disclose a number 1-4 elsewhere.

Supplementary Figure: Timing of COVID-19 infection in the pregnancy cohort


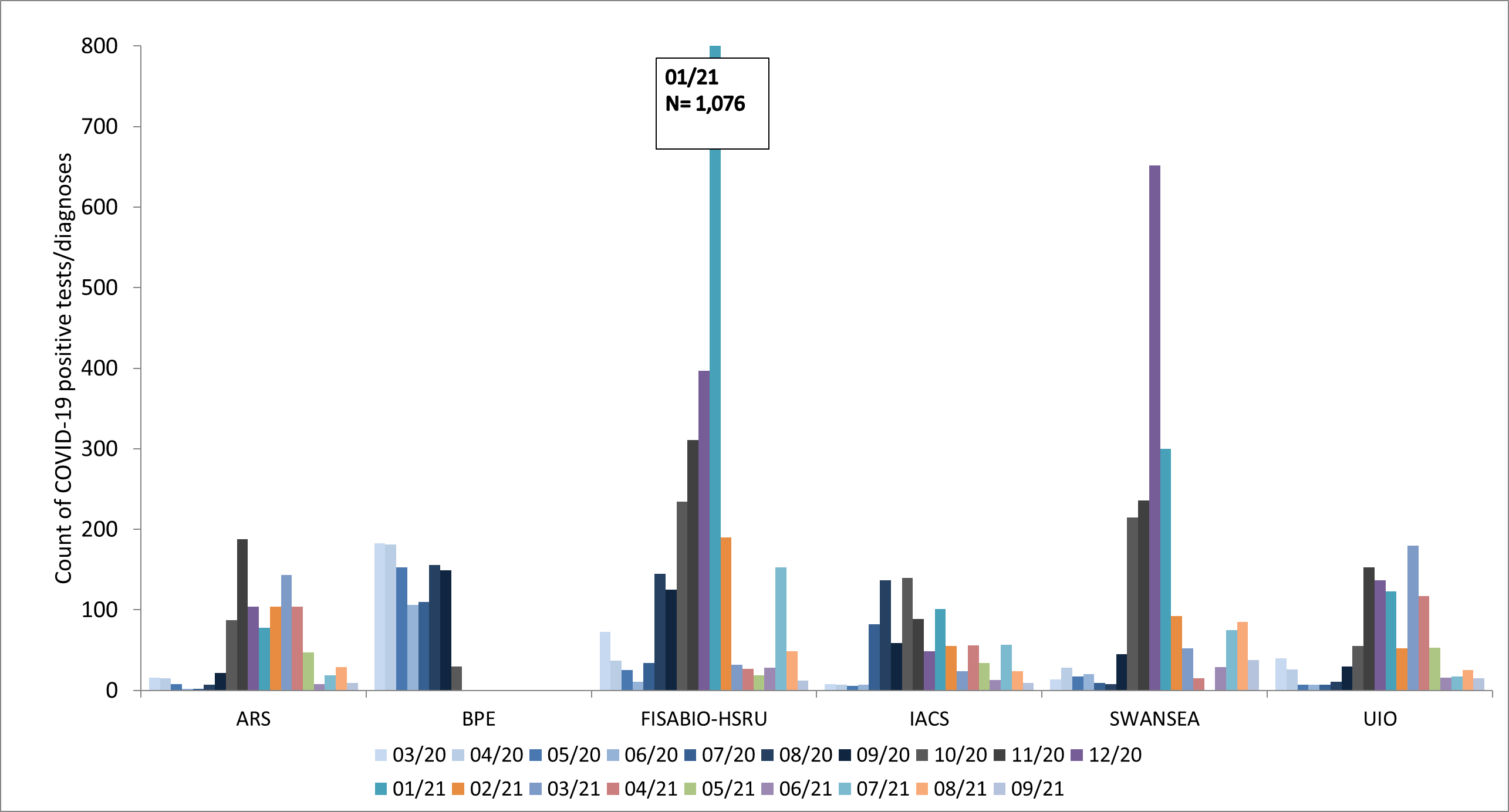


The figure presents calendar time of COVID-19 positive test or disease by month from March 2020 to September 2021 for the cohort of pregnant women who had a positive test or diagnosis for COVID-19 during their pregnancy. Most COVID-19 diagnoses/positive tests occurred between 09/2020 and 03/2021.

Abbreviations: ARS = Agenzia Regionale di Sanita’ della Toscana; BPE = FISABIO-HSRU = Foundation for the Promotion of Health and Biomedical Research of Valencia Region - Health Services Research Unit; IACS = Instituto Aragones de Ciencias de la Salud;; UiO = University of Oslo
